# Reviewer Acknowledgement 2012

**DOI:** 10.1186/1471-2202-14-31

**Published:** 2013-04-15

**Authors:** Deesha A Majithia

**Affiliations:** 1 236 Grays Inn Road, London WC1X 8HB, UK

## Abstract

**Contributing reviewers:**

The editors of *BMC Neuroscience* would like to thank all our reviewers who have contributed to the journal in Volume 13 (2012).

## 

Salim Abdelilah-Seyfried

Germany

Usha Acharya

USA

David Adams

Australia

Deanna Adkins

USA

Fabienne Agasse

USA

Tariq Ahmed

Belgium

Jyrki Ahveninen

USA

Conrad Alano

USA

Kimmo Alho

Finland

Guillermina Almazan

Canada

Manuel Alvarez Dolado

Spain

Karem Alzoubi

Jordan

Julià L Amengual

Spain

Miroslava Anderova

Czech Republic

William Andrews

United Kingdom

Michael Antle

Canada

Toshiyuki Araki

Japan

Regina Armstrong

USA

Robert Astur

USA

Stéphane Auvin

France

Mauhamad Baarine

USA

Frank Baas

Netherlands

Claudio Babiloni

Italy

Claudio Babiloni

Italy

Harvey Babkoff

Israel

Ou Bai

USA

Sandra Bajjalieh

USA

Victoria Bajo Lorenzana

United Kingdom

Harriet Baker

USA

Bryan Ballif

USA

Maxim Bazhenov

USA

Richard Bazinet

USA

Walter Becker

Germany

Peter Bede

Ireland

Richard Bell

USA

Irina Beloozerova

USA

Nada Ben Abdallah

Switzerland

Alexandra Benchoua

France

Alexandra Bendixen

Germany

Federico Bermudez-Rattoni

Mexico

Yasumasa Bessho

Japan

Frederike Beyer

Germany

Massimiliano Bianchi

France

Gregory Bix

USA

Samuel Boateng

United Kingdom

Staffan Bohm

Sweden

Severine Boillee

France

Tatiana Borisova

Ukraine

Jasenka Borzan

USA

Catherine Bowes Rickman

USA

Gregory Brewer

USA

Holly Bridge

United Kingdom

Maximilian Bruchmann

Germany

Norbert Bruggemann

Denmark

Luigi Brunetti

Italy

Thomas Brushart

USA

Barbara Budzynska

Poland

Hugo Cabedo

Spain

Elisa Cabiscol

Spain

Michael Cahill

USA

Ruth Campbell

United Kingdom

Clint Canal

USA

Jose L. Cantero

Spain

Arvind Caprihan

USA

Rudolf Cardinal

United Kingdom

Alan Carleton

Switzerland

Susan Carnell

United Kingdom

Jeffrey Carroll

USA

Patrick Carroll

France

Umberto Castiello

Italy

Enrico Cataldo

Italy

Ana Catarino

United Kingdom

Kathryn Chadman

USA

Thierry Charlier

USA

Marie-Christine Chartier-Harlin

France

Frederic Checler

France

Jeannie Chen

USA

Ling Chen

China

Lu Chen

USA

Raymond Cheung

Hong Kong

Joan Chiao

USA

Sukumal Chongthammakun

Thailand

Lopez Christophe

France

Sean Clark

USA

Marie Cleary

United Kingdom

Carine Cleren

France

Yann Coello

France

Allison Coffin

USA

Olympia Colizoli

Netherlands

Fiorenzo Conti

Italy

Mark S Cooper

USA

John Crabbe

USA

Samuel Crish

USA

Toni Cunillera

Spain

Letizia Da Sacco

Italy

Wouter De Baene

Belgium

Karolien De Bosscher

Belgium

Andrea De Cesarei

Italy

Mariken De Koning

Netherlands

Elvira De Leonibus

Italy

Maarten De Vos

Germany

Stefan Debener

Germany

Nima Dehghani

France

Marc Del Bigio

Canada

Rona Delay

USA

Thomas Demarse

USA

Michele Devido-Santos

Brazil

Vincenzo Di Marzo

Italy

Elva Diaz

USA

Travis Dickendesher

USA

Margarita Diez

Sweden

Hans-Ulrich Dodt

Austria

Nuria Doñamayor Alonso

Germany

Enkui Duan

China

Carlos Duarte

Portugal

Luc Dupuis

France

Alfred Effenberg

Germany

Marc Ekker

Canada

Evangelia Emmanouilidou

Greece

Juergen Engele

Germany

Samuel Erb

USA

Jeffrey Erickson

USA

Lynda Erskine

United Kingdom

David Featherstone

USA

Irwin Feinberg

USA

David Fernandez Lopez

USA

Javier Fernandez-Ruiz

Spain

Liana Fidani

Greece

Paolo Follesa

Italy

Aaron Fox

USA

Bernd Fritzsch

USA

Marcus Fruttiger

United Kingdom

Ken-Ichiro Fukuchi

USA

Nobuyuki Fukushima

Japan

Ml Fung

Hong Kong

Xavier Gallego

USA

Maria Joao Gama

Portugal

Fuqiang Gao

Canada

Fabrizio Gardoni

Italy

Antonella Gasbarri

Italy

Lynne Gauthier

USA

Sherly George

New Zealand

Karen Gertz

Germany

Stefano Geuna

Italy

Othman Ghribi

USA

Bradley Gibson

USA

Nicholas Gilpin

USA

Joel Glover

Norway

Martin Goettlich

Germany

Tim Gollisch

Germany

Flávia Gomes

Brazil

Jose Luis Gonzalez De Aguilar

France

Christian Gonzalez-Billault

Chile

Kristin Gosselink

USA

Vinicio Granados-Soto

Mexico

Oliver Granert

Germany

William Graves

USA

William Green

USA

Grace Griesbach

USA

Darren Griffin

United Kingdom

Boyan Grigorov

France

Raymond Grill

USA

Owen Gross

USA

Nicolas Guerout

France

Jun Guo

China

Hirac Gurden

France

Young Gwak

USA

Hashem Haghdoost-Yazdi

Iran

Matthew Hale

Australia

Anita Hall

United Kingdom

Sagi Harnof

Israel

Theresa Harrison

USA

Richard Hartman

USA

Qiaojun He

China

Ying He

USA

Yong He

China

Ashok Hegde

USA

Urs Heilbronner

Germany

Marcus Heldmann

Germany

Christoph Helmchen

Germany

Michael Hendricks

USA

Matthias H Hennig

United Kingdom

Nils Henninger

USA

David Henshall

Ireland

Pierre-Yves Herve

France

Catherine Hesford

United Kingdom

Constanze Hesse

United Kingdom

Scott Heximer

Canada

Mikko Hiltunen

Finland

Marc Himmelbach

Germany

Kate Himmelmann

Sweden

Ann Hoffman

USA

Ulrich Hofmann

Germany

Helena Hogberg

USA

Markus Höltje

Germany

Jens-Max Hopf

Germany

Yuuichi Hori

Japan

Jean-Christophe Houzel

Brazil

James Howard

USA

Chi-Mei Hsueh

Taiwan

Bingren Hu

USA

Jason Huang

USA

Rong-Chi Huang

Taiwan

Yao Huang

USA

Andrea Huber

Germany

Richard Hume

USA

Sandrine Humez

France

Christian Humpel

Austria

Xia Huo

China

Fatima Husain

USA

Yasuyuki Igarashi

Japan

Masafumi Ihara

Japan

Peter Indefrey

Germany

Yasuki Ishizaki

Japan

Mbemba Jabbi

USA

Ingrid Jakob

France

Nihar Jana

India

Erich Jarvis

USA

Norbert Jausovec

Slovenia

Adrianna Jenkins

USA

Poul Henning Jensen

Denmark

Vesna Jevtovic-Todorovic

USA

Jack Jhamandas

Canada

Yongfeng Jin

China

Dan-Anders Jirenhed

Sweden

Ray Johnson

USA

Rex Jung

USA

Giedrius Kalesnykas

Finland

Helen Kamens

USA

Henry Kaminski

USA

Bjoern Kampa

Switzerland

Nancy Kanagy

USA

Shigeaki Kanatani

Sweden

Takashi Kanbayashi

Japan

Norio Kaneda

Japan

Kenji Kansaku

Japan

Panagiotis Kassavetis

United Kingdom

Jun-Ya Kato

Japan

Hiroshi Katsuki

Japan

Matthieu Keller

France

Ilan Kerman

USA

Nicoletta Kessaris

United Kingdom

Sarah Ketay

USA

William Killgore

USA

Chobok Kim

Korea South

Juno Kim

Australia

Markus Kipp

Germany

Tilmann Alexander Klein

Germany

Anna Klintsova

USA

Christian Kluge

Germany

Antti Knaapila

Finland

Maria Knikou

USA

Verner Knott

Cape Verde

Jane Ko

USA

Jian Kong

USA

Sergiy M Korogod

Ukraine

Sören Krach

Germany

Ulrike Krämer

Germany

Mariska Kret

Netherlands

James Krueger

USA

Diana Kunze

USA

Masashi Kurachi

Japan

Tomoyuki Kuwaki

Japan

John Kwok

Australia

Peter Lackner

Austria

Zdravko Lackovic

Croatia

Ayelet Lahat

Canada

Joseph Lamanna

USA

Raphael Lamprecht

Israel

Emma Lane

United Kingdom

Bing Lang

United Kingdom

Charles Larson

USA

Jeanne Latourelle

USA

Alpar S Lazar

United Kingdom

Philip Lazarovici

Israel

Colleen Le Prell

USA

Hao Lei

China

Jean-Francois Lepage

USA

Noelle L'Etoile

USA

Leonard Levin

USA

Andrew Levine

USA

Steve Levison

USA

Jonathan Levitt

USA

Fengqiao Li

USA

Yifan Li

USA

Zheng Li

USA

Dieter Chichung Lie

Germany

Hsiao-Wen Lin

Taiwan

Longnian Lin

China

Ming-Fong Lin

USA

Omer Linkovski

Israel

Shoei-Yn Lin-Shiau

Taiwan

Patricia Lisboa

Brazil

Nai-Kui Liu

USA

Qiang Liu

USA

Yijun Liu

USA

Ann Logan

United Kingdom

Julian Lombard

USA

Joan Lopez-Moliner

Spain

Yong Lu

USA

Albert Ludolph

Germany

David Luque

Spain

Yuanye Ma

China

Robert Macdonald

Canada

Alan Mackay-Sim

Australia

Panchanan Maiti

India

Raghunath Manchala

India

Francois Marceau

Canada

Veronique Marchand-Pauvert

France

Mathieu Marella

USA

Ann Marini

USA

Kirill Martemyanov

USA

Laura Martin

USA

Salvador Martinez

Spain

Alberto Martire

Italy

Grant Mastick

USA

Klaus Mathiak

Germany

Tomohiro Matsuyama

Japan

Doug Matthews

Singapore

Martin H. Maurer

Germany

Victor May

USA

Jodi Mcbride

USA

Dana Mctigue

USA

Bruno Meloni

Australia

Charles Meshul

USA

Adrian Michael

USA

Silvia Middei

Italy

Neil Millar

United Kingdom

Janet Miller

USA

Diego Minciacchi

Italy

Mirko Miuzzi

Italy

Edwin Monuki

USA

Teresa Morales

Mexico

Caroline Moreau

France

Laura Morelli

Argentina

Nadia Mores

Italy

Hitoshi Morikawa

USA

Emmanuel Moyse

France

Khyobeni Mozhui

USA

William Mundy

USA

Junko Murata

Japan

Juan Nacher

Spain

Takayuki Nakagawa

Japan

Harukazu Nakamura

Japan

Keiko Nakanishi

Japan

Winfried Neuhaus

Austria

Donald Francis Newgreen

Australia

Laurent Nguyen

Belgium

Charles Nichols

USA

Ellen Niederberger

Germany

Sander Nieuwenhuis

Netherlands

Lylia Nini

Canada

Andrea Nistri

Italy

Michael Nitsche

Germany

Maria Nolano

Italy

Angel Nunez

Spain

K. Elisabet Ohlin

Sweden

Shigeki Ohta

Japan

Hiroyuki Okuno

Japan

Foster Olive

USA

Alexandre Leite Rodrigues Oliveira

Brazil

Etienne Olivier

Belgium

Doreen Olvet

USA

Yumie Ono

Japan

John Mark Pabona

USA

Daniel Pak

USA

Adrian Parry-Jones

United Kingdom

Giulio Pasinetti

USA

Luca Passamonti

Italy

Jeroen Pasterkamp

Netherlands

William Pearce

USA

Domenico E. Pellegrini-Giampietro

Italy

Dominic Pérennou

France

Francesc Perez-Branguli

Germany

Brian Perkins

USA

Juliana Perry

Brazil

Nicolas Caesar Petersen

Denmark

Jeffrey Petruska

USA

Michele Pierro

USA

Graham Pitcher

Canada

Celine Plachez

USA

Robert Plomin

United Kingdom

Steven Pollard

United Kingdom

Igor Pomytkin

Russian Federation

Aurel Popa-Wagner

Germany

Paul Pope

United Kingdom

Paula Pousinha

Portugal

Elizabeth Powell

USA

Christine Preibisch

Germany

Hubert Preissl

Germany

Ashley Pringle

United Kingdom

Irina Prots

Germany

Julien Puyal

Switzerland

Bin Qin

China

Jean-Francois Quignard

France

John Quindry

USA

Elizabeth Race

USA

Eugenia Radulescu

United Kingdom

Rohit Ramchandra

Australia

Ami Raval

USA

Vijayalakshmi Ravindranath

India

Eva Redei

USA

John Redell

USA

Benjamin Reese

USA

Johannes M.H.M. Reul

United Kingdom

Heather Richardson

USA

William Richardson

United Kingdom

Francisco J. Rivera

Austria

Alan Roberts

United Kingdom

Craig Roberts

United Kingdom

Borja Rodríguez

Spain

Karin Rosenkranz

Germany

Alan Rosenwasser

USA

Simone Rossi

Italy

Paula Row

United Kingdom

Pierre-Alain Rubbo

France

Eric Rubinstein

France

Jascha Ruesseler

Germany

Elena Rugarli

Italy

Emilio Russo

Italy

Elena Saftenku

Ukraine

Sanjeev Sahni

USA

Hiroyuki Sakagami

Japan

Harutoshi Sakakima

Japan

Carlo Sala

Italy

Tarek Saleh

Canada

Devadoss Samuvel

USA

Farahnaz Sananbenesi

Germany

Pascal Sanchez

USA

Jose Sanchez-Mut

Spain

Ursula Sandau

USA

Carmen Sandi

Switzerland

Monica Santos

Spain

Carlos Saura

Spain

Nic Savaskan

Germany

Johannes Schlachetzki

Germany

Gottfried Schlaug

USA

Susanne Schmid

Canada

Thomas Schmitz

Germany

Henrike Scholz

Germany

Andreas Schulze

Canada

Antonella Scorziello

Italy

Tim Seabrook

Switzerland

Philippe Seguela

Canada

Armin Seidl

USA

Daniel Senkowski

Germany

Stefano Sensi

USA

Nicolas Sergeant

France

Laurent Servais

France

Emily Severance

USA

James Shaffery

USA

Christopher Shaw

Canada

Jun-Xian Shen

China

Koji Shibasaki

Japan

Takayoshi Shimohata

Japan

Derya Shimshek

Switzerland

Fabricio Simao

Brazil

Ishwar S Singh

USA

Anna-Leena Sirén

Germany

Luigi Sironi

Italy

Joel Snyder

USA

Farida Sohrabji

USA

Young-Jin Son

USA

Yan Song

China

João Sousa

Portugal

Paul Sowman

Australia

Michael Spratling

United Kingdom

Katja Spreckelmeyer

Germany

Andreas Sprenger

Germany

James St John

Australia

Paul Stapley

Australia

Jena Steinle

USA

Mitchell Steinschneider

USA

Markus Stevens

Netherlands

Ryan Stevenson

USA

Maja Stikic

Serbia

Cathy Stinear

New Zealand

George Stoica

USA

Irina Stoyanova

Netherlands

Frank Striggow

Germany

Yi-Huang Su

Germany

Xi-Qing Sun

China

Makoto Suzuki

Japan

Yuichiro Suzuki

USA

Josef Syka

Czech Republic

Gregor R. Szycik

Germany

Hiroshige Takeichi

Japan

Jussi Tallus

Finland

Hideaki Tanaka

Japan

Lei Tang

USA

Jean-Marc Taymans

Belgium

Takeshi Terao

Japan

Elvar Theodorsson

Sweden

Jean-Leon Thomas

USA

Mario Tombini

Italy

Gonzalo Torres

USA

Yvonne Tran

Australia

Peter Trillenberg

Germany

Vincent Tropepe

Canada

Sascha Tyll

Germany

Esther Udina

Spain

Yashimoto Ueda

Japan

Kathrin Utz

Germany

Gilles Vadewalle

Belgium

Frank Van Bel

Netherlands

Maarten Van Den Buuse

Australia

Lisette Van Der Meer

Netherlands

Michiel Van Elk

Switzerland

Peter Vanhoutte

France

Alfredo Varela-Echavarria

Mexico

Christopher Vaughan

Australia

Demetrios Vavvas

USA

Antonio Velayos

United Kingdom

Claudia Verderio

Italy

Anthony Vernon

United Kingdom

Marcia Vilela

Brazil

Valerie Vingtdeux

USA

Jaakko Virtanen

Finland

Vladimir Vladimirov

USA

Gregor Volberg

Germany

David Volk

USA

Cinzia Volonte'

Italy

Malcolm Von Schantz

United Kingdom

Eero Vuoksimaa

Finland

Ajai Vyas

Singapore

Christine Wagner

USA

Daniel-Christoph Wagner

Germany

Adam Walker

USA

Matthew Walsh

USA

Francisco Wandosell

Spain

Gang Wang

USA

Qing Wang

China

Wei Wang

China

Eric Warrant

Sweden

Gerald Watson

USA

Béla Weiss

Hungary

Marie-Laure Welter

France

Shih-Jen Weng

Singapore

Wolfgang Weninger

Austria

Stig Wergeland

Norway

Hauke Werner

Germany

Carmen Westerberg

USA

Stephanie White

USA

Marlene Wiart

France

Jamie Wilkinson

United Kingdom

Roel Willems

Netherlands

Justin Williams

United Kingdom

Ursula Winzer-Serhan

USA

Oliver Wirths

Germany

Thomas Wisniewski

USA

Daniel Wiswede

Germany

Karsten Witt

Germany

George Wittenberg

USA

Kelvin Wong

USA

Georgia Woods

USA

Di Wu

China

Guoxin Wu

USA

Ye Xiong

USA

Lin Xu

China

Pingyi Xu

China

Emre Yaksi

Belgium

Fulya Yalcinkaya

Turkey

Masahiro Yamaguchi

Japan

Bryan Yamamoto

USA

Bo Yang

China

Chae Ha Yang

Korea South

Mu Yang

USA

Shaohua Yang

USA

Masahiro Yasuda

USA

Midori Yenari

USA

Ozgur Yilmaz

Turkey

Hao-Jun You

China

Wing-Ho Yung

Hong Kong

Tino Zaehle

Germany

Marta Zagrebelsky

Germany

Chenggang Zhang

China

Jie Zhang

USA

John Zhang

USA

Yun Zhang

USA

Chunlin Zhao

China

Chunmei Zhao

USA

Heng Zhao

USA

David Zilles

Germany

Luc Zimmer

France

Lauryn Zipse

USA

Liga Zvejniece

Latvia

